# Temporal lobe epilepsy alters neural responses to human and avatar facial expressions in the face perception network

**DOI:** 10.1002/brb3.2140

**Published:** 2021-05-05

**Authors:** Lorena Chantal Kegel, Sascha Frühholz, Thomas Grunwald, Dieter Mersch, Anton Rey, Hennric Jokeit

**Affiliations:** ^1^ Swiss Epilepsy Center Zurich Switzerland; ^2^ Department of Psychology University of Zurich Zurich Switzerland; ^3^ Institute for Critical Theory Zurich University of the Arts Zurich Switzerland; ^4^ Institute for the Performing Arts and Film Zurich University of the Arts Zurich Switzerland

**Keywords:** avatar, emotion, facial expression, perception, temporal lobe epilepsy

## Abstract

**Background and Objective:**

Although avatars are now widely used in advertisement, entertainment, and business today, no study has investigated whether brain lesions in neurological patients interfere with brain activation in response to dynamic avatar facial expressions. The aim of our event‐related fMRI study was to compare brain activation differences in people with epilepsy and controls during the processing of fearful and neutral dynamic expressions displayed by human or avatar faces.

**Methods:**

Using functional magnetic resonance imaging (fMRI), we examined brain responses to dynamic facial expressions of trained actors and their avatar look‐alikes in 16 people with temporal lobe epilepsy (TLE) and 26 controls. The actors' fearful and neutral expressions were recorded on video and conveyed onto their avatar look‐alikes by face tracking.

**Results:**

Our fMRI results show that people with TLE exhibited reduced response differences between fearful and neutral expressions displayed by humans in the right amygdala and the left superior temporal sulcus (STS). Further, TLE was associated with reduced response differences between human and avatar fearful expressions in the dorsal pathway of the face perception network (STS and inferior frontal gyrus) as well as in the medial prefrontal cortex.

**Conclusions:**

Taken together, these findings suggest that brain responses to dynamic facial expressions are altered in people with TLE compared to neurologically healthy individuals—regardless of whether the face is human or computer‐generated. In TLE, areas sensitive to dynamic facial features and associated with processes relating to the self and others are particularly affected when processing dynamic human and avatar expressions. Our findings highlight that the impact of TLE on facial emotion processing must be extended to artificial faces and should be considered when applying dynamic avatars in the context of neurological conditions.

## Graphical abstract

The aim of our event‐related fMRI study was to compare brain activation differences in people with epilepsy and controls during the processing of fearful and neutral dynamic expressions displayed by human or avatar faces. Our fMRI results show that people with TLE exhibited reduced response differences between human and avatar fearful expressions in the dorsal pathway of the face perception network (STS and inferior frontal gyrus) as well as in the medial prefrontal cortex. Taken together, these findings suggest that brain responses to dynamic facial expressions are altered in people with TLE compared to neurologically healthy individuals—regardless of whether the face is human or computer‐generated.

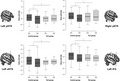

## 1 INTRODUCTION

Advances in the development and animation of computer‐generated characters have led to the increased usage of anthropomorphic characters in digital applications and communication technologies (Miller, 2007). Accordingly, computer‐generated characters, or avatars, have also become popular for clinical and research settings as a complement to existing communication, assessment, and therapy options (Bohil et al., 2011; Bombari et al., 2015). As such, there have been initial studies examining the use of human‐like avatars in the assessment and training of patients with neurological conditions (Aljaroodi et al., 2017; Boucenna et al., 2014; Georgescu et al., 2014; Javor et al., 2016; Robitaille et al., 2017; Schilbach et al., 2007). When avatars are used in such settings, they—like humans—can accompany and influence interactions with facial expressions and thereby transmit social information. However, we do not know how flexibly we react to and integrate virtual non‐conspecifics into our social environment. What are the costs in terms of intensity and effort of emotional exchange in human‐avatar interactions compared to interactions between humans? This missing knowledge together with the increased exposure to avatars motivates the present investigation of the perception of humans and avatars. In particular, it is unclear how mesial temporal brain areas that play an eminent role in the processing of affective stimuli respond to these newly existing interaction partners. For this reason, it is essential to investigate whether lesions within the temporal lobe, such as those exhibited by individuals with temporal lobe epilepsy (TLE), impact the response. Determining how human and avatar faces are processed in the brain when TLE is present may provide significant insights about the importance of the affected brain regions. Hence, in the present study, we investigate whether brain responses to dynamic expressions displayed by human and avatar faces differ between people with TLE and neurologically healthy people.

In TLE, lesions in the amygdala, the hippocampus, or lateral temporal areas are associated with extensive structural and functional alterations in the temporal lobe and extratemporal regions such as frontal cortex (Bernhardt et al., 2013; Engel & Salamon, 2015; Jokeit et al., 1997). These changes encompass the network that is engaged during facial emotion perception and could thus be associated with impairments in the processing and recognition of emotions in people with TLE (Ives‐Deliperi & Jokeit, 2019; Jokeit et al., 2018; Milesi et al., 2014; Monti & Meletti, 2015; Schacher, Winkler, et al., 2006). In this neural face perception network, the superior temporal sulcus (STS) and the inferior frontal gyrus (IFG) belong to the dorsal pathway, which is sensitive to dynamic facial features such as facial motion and gaze. In addition, the inferior occipital gyrus (IOG), the fusiform gyrus (FG), and the anterior temporal lobe (ATL) form the ventral pathway sensitive to invariant facial features such as form and configuration. Moreover, the amygdala plays a central role in the processing of emotional facial expressions by contributing to the fast detection and evaluation of salient signals in our environment (Adolphs, 2001; LeDoux, 2000). This role is highlighted by amygdalar feedback connections to the dorsal and ventral pathway in the face perception network, which enable a modulatory effect on cortical face processing (Furl et al., 2013; Haxby et al., 2000; Vuilleumier, 2005). Together, this extended network forms the neural basis for the processing of facial expressions and facial identity (Duchaine & Yovel, 2015; Haxby et al., 2000).

In line with the above‐mentioned findings, previous research has reported that people with TLE show altered activity in face‐sensitive cortical and subcortical areas in response to human facial expressions. Accordingly, it has been shown that people with TLE displayed smaller responses in the amygdala, the occipital fusiform gyrus, the FG, and the posterior part of the STS than controls in response to dynamic fearful expressions (Åhs et al., 2014; Ives‐Deliperi et al., 2017; Labudda et al., 2014; Riley et al., 2015; Schacher, Haemmerle, et al., 2006; Toller et al., 2015; Vuilleumier et al., 2004). Furthermore, people with TLE showed extensive alterations of functional connectivity in distributed areas subserving facial emotion processing in contrast to controls (Broicher et al., 2012; Riley et al., 2015; Steiger & Jokeit, 2017). This highlights the importance of the affected regions in TLE and their influence on the whole‐brain network subserving facial emotion processing (Ives‐Deliperi & Jokeit, 2019).

Based on the evidence reported above, we may conclude that there are differences regarding the processing of dynamic human expressions between people with and without TLE. However, no previous study has tested whether response differences in the amygdala and the face perception network also translate to the processing of dynamic expressions of avatars. Recent evidence with neurologically healthy individuals suggests that areas in the dorsal pathway of the face perception network show stronger responses to human expressions than to avatar expressions. This has been shown for the STS and the IFG that are sensitive to dynamic features of faces and thus may show stronger responses to natural facial motion than to artificial facial motion (Duchaine & Yovel, 2015; Haxby et al., 2000; James et al., 2015; Kätsyri et al., 2020; Kegel et al., 2020; Sarkheil et al., 2013). Further, differences between dynamic human and avatar faces have so far only been reported for fearful expressions and not for neutral expressions (Kegel et al., 2020). Based on this, we may assume that emotional expressions exert a significant influence on human and avatar face processing in dorsal temporal areas, possibly via amygdalar‐cortical feedback connections (Furl et al., 2013). How TLE and associated structural and functional alterations in the temporal lobe and beyond may further affect this processing is unknown.

Hence, in the current study, we examined response differences to dynamic human and avatar expressions in people with TLE and controls with whole‐brain fMRI. Drawing on previous findings of the processing of human faces in TLE, we hypothesized that people with TLE would show overall attenuated brain responses to fearful human expressions versus neutral human expressions when compared to controls. We expected this response pattern to be present in dorsal and ventral areas of the face perception network as well as in the amygdala. Regarding response differences between human and avatar expressions, we expected that structural and functional alterations in people with TLE would affect the processing of both stimulus types. Therefore, we assumed a smaller response difference between human and avatar expressions for people with TLE compared to controls. Taking into account previous results with avatar faces, we assumed that such group differences would mainly occur in dorsal areas of the face perception network sensitive to dynamic features of faces.

## 2 MATERIALS AND METHODS

### 2.1 Sample

We examined 17 people with TLE and 30 controls that reported no diagnosed psychiatric or neurological disorders. People with TLE were recruited at the Swiss Epilepsy Center in Zurich. The main inclusion criterion was focal seizures originating in one or both temporal lobes. This criterion had been confirmed by ictal video‐EEG and the seizure type recorded during previous in‐patient stays at the center. In two people with TLE, this criterion was confirmed by interictal EEG and the seizure type reported by the affected person and/or an eyewitness, as both had not been examined as inpatients. Consequently, it was not possible to lateralize the seizure origin in these two people with TLE and both were only included for analyses of activation differences between the control group and the entire TLE group (regardless of seizure origin, see Section 2.4). The TLE diagnoses were made by epileptologists at the Swiss Epilepsy Center.

The control group was recruited via online advertising on a local community website and in‐house advertising targeted at the staff of the Swiss Epilepsy Center. All participants had to be able to follow and understand the information and study procedure (i.e., no language barrier, severe cognitive deficit, or psychiatric disease). Their vision was required to be normal or corrected to normal and all participants had to fulfill standard MRI safety criteria. All procedures as well as the study design were approved by the local ethics committee and participants were tested only following written informed consent in accordance with the Declaration of Helsinki.

During the preprocessing of the data (see Section 2.4), we had to exclude one participant from the TLE group due to severe atrophy of the left brain hemisphere which caused the preprocessing to fail. From the control group, two participants had to be excluded from final analyses due to excessive movement (>2 mm in either *x*‐, *y*‐, or *z*‐direction), one due to insufficient task engagement (verified by our control task described in Procedure and Stimuli), and one due to discomfort that led to the termination of the scanning session. This resulted in a sample of 16 people with TLE and 26 controls. Please see Table 1 for sociodemographic and clinical characteristics of the sample and Section 3.1 for analysis of group differences.

**TABLE 1:** Sociodemographic and clinical characteristics of participants with and without temporal lobe epilepsy

	Control group *n* = 26	TLE group *n* = 16	Left TLE group *n* = 7	Right TLE group *n* = 7
Sociodemographic characteristics				
Gender (m/f)	13/13	4/12	2/5	2/5
Age in years, *Mdn* (range)	38.6 (18–62)	47.6 (21–64)	48.4 (21–58)	47.8 (28–64)
Years of full‐time education, *Mdn* (range)	13 (9–21)	12 (9–15)	12 (9–15)	13 (10–14)
Clinical characteristics				
Age at epilepsy onset in years, *Mdn* (range)		22.5 (5–62)	17 (6–35)	22 (5–62)
Duration of epilepsy in years, *Mdn* (range)		20.5 (2–42)	27 (7–42)	19 (2–35)
Number of antiepileptic drugs per day, *Mdn* (range)		2 (0–3)	2 (0–3)	2 (1–2)
Hippocampal sclerosis (yes/no)		11/5	7/0	4/3

Note

The analyses of group differences regarding the sociodemographic and clinical characteristics were not significant (all *p* > .05).

Abbreviations: f, female; m, male; Mdn, median; TLE, temporal lobe epilepsy.

### 2.2 Procedure and stimuli

**FIGURE 1:**
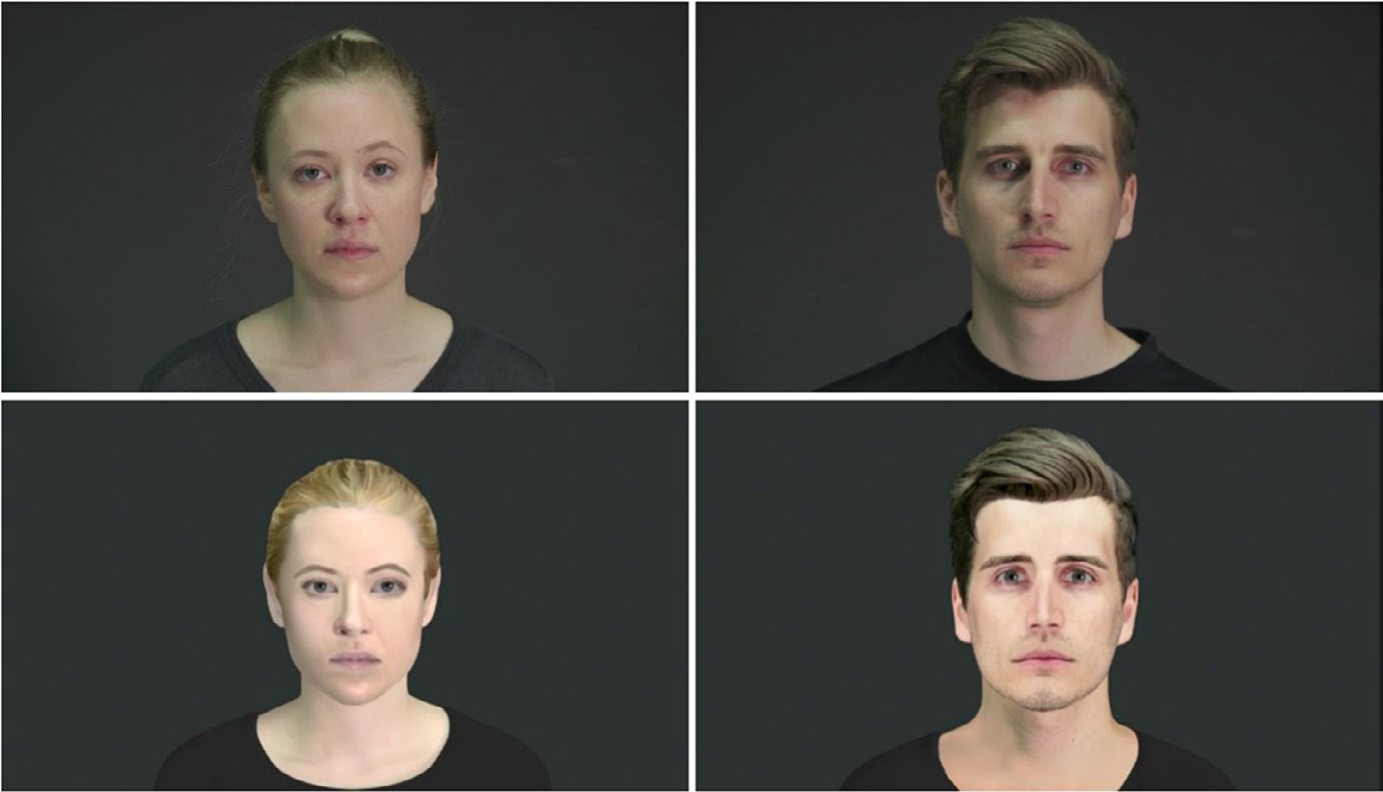
Illustration of a female and a male actor (top panels) and their corresponding avatars (bottom panels) displaying neutral expressions

We used an event‐related fMRI protocol presenting videos of actors' facial expressions and their avatar look‐alikes to measure blood‐oxygen‐level‐dependent (BOLD) responses associated with facial emotion processing. Participants completed 208 trials with videos of human and avatar faces showing fearful and neutral expressions, as well as scrambled versions of these videos (see Figure 1 and Videos S1 and S2 online). Furthermore, control videos with a red square centered on the displayed face or scrambled pattern were infrequently presented to which participants had to respond with a button press. The 208 trials were divided into two runs, so that each run consisted of 32 videos of human expressions (16 each fearful and neutral), 32 videos of avatar expressions (16 each fearful and neutral), 32 scrambled videos, and 8 control videos.

The videos lasted 3 s and were randomly presented with Cogent 2000 (version: 1.32; http://www.vislab.ucl.ac.uk/cogent.php, RRID: SCR_015672) under MATLAB (version 2015a; https://ch.mathworks.com/products/matlab.html; RRID: SCR_001622). In the scanner room, the videos were projected onto a projection screen situated at the front of the MRI bore (image width 103.5 cm, image height 85.5 cm). Participants were able to see the videos on the projection screen through a mirror attached to the head coil (visual angle of the faces: 7° (horizontal), 8.5° (vertical)). A Panasonic LCD Projector with wide angle optics, a screen resolution of 1,024 × 768 pixels, a brightness of 1,200 lm, and a frame rate of 50 Hz was used.

Participants were instructed to watch the videos attentively and to respond with a button press if a video with a red square was presented. The total number of button presses and the response times were recorded, so that participants' task engagement was verified. After scanning, participants were reimbursed with 30 Swiss Francs. Furthermore, participants were informed that they would be asked to rate the videos of human and avatar expressions according to their intensity in an online rating survey. Each survey contained 32 videos showing fearful human and avatar expressions (16 each) and four videos showing neutral human and avatar expressions (2 each) as a control condition. The intensity rating of the facial expressions could range from 1 (*not very intense*) to 6 (*extremely intense*) and had to be completed within 2 weeks.

The study protocol and data from controls were part of a previous analysis described in a published work by our group. For more details regarding the development of the videos displaying human and avatar expressions and the intensity rating, please refer to Kegel et al., (2020).

### 2.3 MRI data acquisition

All MRI data were collected using a 3 Tesla Philips Achieva scanner (Philips Medical Systems) with a 32‐channel head coil. Anatomical images were collected using a T1‐weighted MPRAGE sequence covering the whole brain and the following scanning parameters: TR/TE = 8.1 ms/3.7 ms, slices = 176 sagittal slices, voxel size = 1 × 1 × 1 mm, matrix size = 240 × 164 mm, FOV = 240 × 240 mm, flip angle = 8, no fat suppression, total acquisition time = 05:37. Functional images were acquired with an EPI sequence with 32 sequential ascending axial slices co‐planar to the AC‐PC line (TR/TE = 1,800 ms/30 ms, voxel size = 2.75 × 2.75 × 3.5 mm, interslice gap = 0.4 mm, matrix size = 80 × 82 mm, FOV = 222 × 222 mm, flip angle = 75, total acquisition time = 14:22). Per run, the first 10 volumes were discarded to allow the equilibration of T1 saturation effects so that in total 467 volumes were acquired.

### 2.4 Imaging preprocessing and analysis

Imaging preprocessing was carried out with SPM12 (version 6906; http://www.fil.ion.ucl.ac.uk/spm/; RRID: SCR_007037) on MATLAB (version 2017a; https://ch.mathworks.com/products/matlab.html; RRID: SCR_001622). Functional images were realigned to the first image in the series, followed by slice timing to the middle slice, and coregistration of the mean functional image to the individual anatomical image. Next, the anatomical scans were segmented into different tissue types and spatially normalized to the Montreal Neurological Institute template using DARTEL (Ashburner, 2007). Simultaneously, a mean anatomical template for the whole group was generated. Functional images were then resampled at a resolution of 2 × 2 × 2 mm and spatially smoothed (8 mm full‐width at half‐maximum Gaussian kernel) to reduce noise.

In the first‐level analysis, individual trials were modeled using a general linear model and the SPM12 default canonical hemodynamic response function defined by the onset and the duration of the videos. All images were high‐pass filtered (cut off 128 s) and the following conditions were modeled as regressors of interest: Condition face type (Human > Avatar), condition facial expression (Fear > Neutral), and condition scramble (nonscrambled > scrambled). Control trials were also modeled as regressors of interest but excluded for second‐level analyses, whereas realignment parameters were included as regressors of no interest.

In the second‐level analysis, we analyzed first‐level contrast images within independent regions of interest (ROI) that have been chosen a priori based on previous literature. Using a probabilistic atlas with particular reference to the temporal lobe (Hammers et al., 2003), we defined the following ROIs: the fusiform gyrus (FG), the posterior superior temporal sulcus (pSTS), the anterior superior temporal sulcus (aSTS), the inferior frontal gyrus (IFG), and the amygdala. Within these regions, average ROI signal was extracted and compared for the different conditions with two‐sample *t*‐tests (Control group > TLE group; TLE group > control group; right TLE > left TLE; left TLE > right TLE) using MarsBaR (version 0.44; http://marsbar.sourceforge.net/index.html; RRID: SCR_009605).

For comparisons between the control group and the TLE group, we pooled the data across participants with TLE to achieve greater statistical power to detect differences. Median rating differences between human and avatar faces were included as covariates of no interest in all analyses, as fearful human expressions were rated as more intense than fearful avatar expressions (see Section 3.1). The resulting two‐sample *t*‐test outcomes in the ROIs were considered significant if they were below *p* < .05. We report an uncorrected threshold (e.g., uncorrected for the number of regions in the ROI analysis) because Bonferroni's adjustment for multiple comparisons is often considered too conservative (Field, 2009). To detect potential group differences outside the a priori defined ROIs, we also analyzed first‐level images over the whole brain for the different contrasts. Regarding these results, we report BOLD activation clusters bigger than a cluster extent of *k* = 5 and remaining significant below a voxel‐wise FWE corrected *p*‐value of <.05.

### 2.5 Analysis of sample characteristics and behavioral data

Before analyzing sample characteristics and the intensity ratings, the respective data distributions were first visually inspected using boxplots. This visual inspection showed that most of the examined variables were not normally distributed. For this reason, between‐group comparisons were performed with Mann–Whitney *U*‐tests. Regarding intensity rating differences, we first compared group differences separately for ratings of fearful human and avatar expressions. Second, we investigated median differences between ratings of fearful human and avatar expressions pooled across the control group and the TLE group. All statistical analyses were performed using SPSS (Version 23; https://www.ibm.com/products/spss‐statistics; RRID: SCR_002865).

## 3 RESULTS

### 3.1 Sample characteristics

People with TLE did not differ significantly from controls in terms of age or years of full‐time education (all *p* > .05). People with right TLE did not differ from those with left TLE concerning age, years of full‐time education, age at epilepsy onset, duration of epilepsy, or number of antiepileptic drugs (all *p* > .05). Further, no significant differences were found between patients with hippocampal sclerosis and patients without hippocampal sclerosis in terms of their clinical characteristics (all *p* > .05). Please see Table 1 for details regarding sociodemographic and clinical characteristics of the sample.

### 3.2 Behavioral data

To verify participants' task engagement during the scanning session, they were required to respond with a button press to infrequently presented control videos with a red square centered on the displayed face or scrambled pattern. The average detection rate of control videos was near perfect in all groups (*M* = 98%–99%). The TLE group (*Mdn *= 772 ms) did not differ from the control group regarding their median response time (*Mdn* =* *769 ms; Mann–Whitney *U*‐test: *U* = 191.000, *p* =* *.673).

**FIGURE 2:**
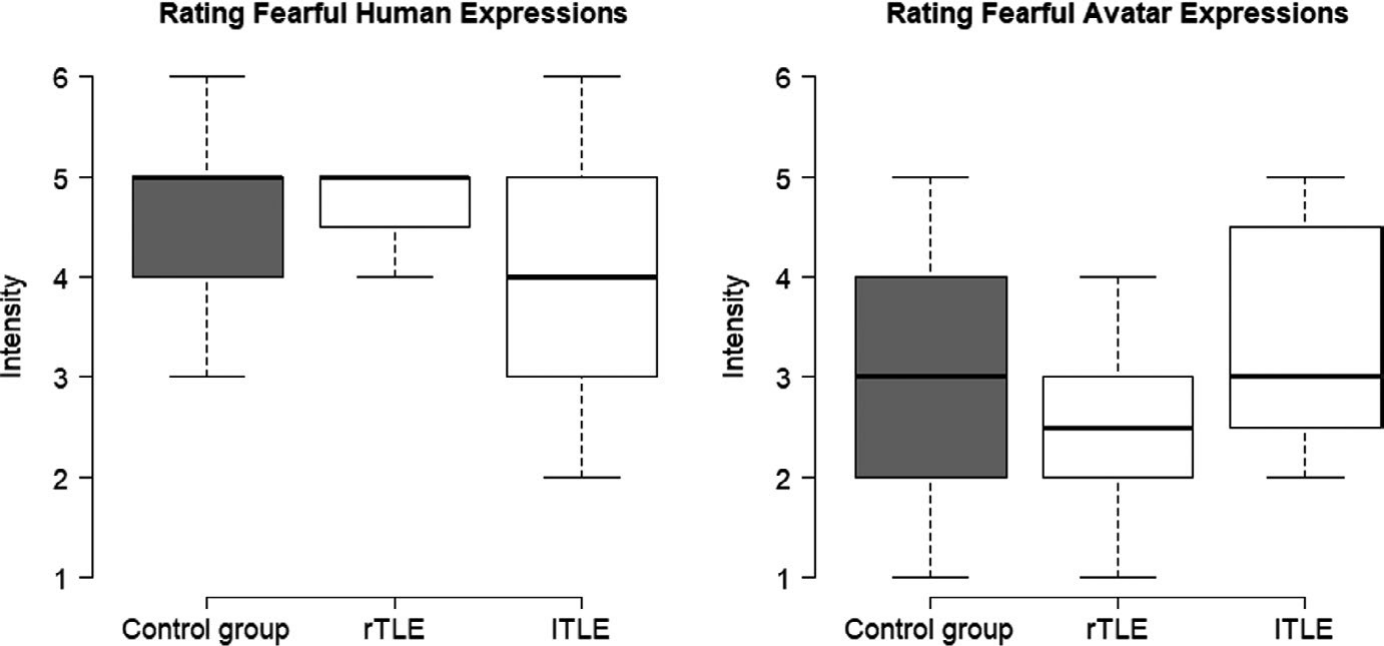
Box plots showing median intensity ratings for fearful human and avatar expressions per group. Whiskers indicate the 25th and 75th percentile. LTLE, left temporal lobe epilepsy; rTLE, right temporal lobe epilepsy

No group differences were found between people with TLE and controls in their intensity rating of fearful human expressions (Mann–Whitney *U*‐test: *U* = 188.000, *p* =* *.593) or their intensity rating of fearful avatar expressions (Mann–Whitney *U*‐test: *U* = 195.500, *p* =* *.808). People with right TLE did not differ significantly from those with left TLE in their intensity rating of fearful human expressions (Mann–Whitney *U*‐test: *U* = 18.000, *p* =* *.462) or their intensity rating of fearful avatar expressions (Mann–Whitney *U*‐test: *U* = 14.500, *p* =* *.240). When comparing fearful human and avatar expressions over the control group and the TLE group, fearful human expressions were rated as more intense (*Mdn* =* *5) than fearful avatar expressions (*Mdn* = 3; Wilcoxon singed‐rank test: *z* =* *−3.63, *p* =* *<* *.001). Please see Figure 2 for the distribution of intensity ratings per group.

### 3.3 BOLD responses to human facial expressions in the extended face perception network

In the control group, fearful versus neutral human expressions evoked greater activation in almost all a priori defined ROIs (FG, pSTS, aSTS, IFG, AMY) except for the left pSTS and the left FG. In people with right TLE, a stronger response to fearful human expressions than to neutral human expressions was found in the right FG, the left aSTS, and bilateral amygdala. Further, people with left TLE did not exhibit a significantly stronger response to fearful human expressions than to neutral human expressions in any of the ROIs (see Table 2 for within‐group statistics). To examine whether this lack of activation difference indicates a lack of activation for people with left TLE in general, we also analyzed the response difference between fearful human expressions and their scrambled counterparts. In this case, people with left TLE showed a stronger response to fearful human expressions than to scrambled expressions in the right amygdala (*t* = 3.35, *p* =* *.003) and bilateral IFG (left: *t* = 3.05, *p* =* *.005; right: *t* = 2.65, *p* =* *.011).

**TABLE 2:** Statistics for the contrast human fearful expression > human neutral expression within each group and for each a priori defined region of interest in the extended face perception network

	FG		pSTS		aSTS		IFG		AMY	
	Left	Right	Left	Right	Left	Right	Left	Right	Left	Right
Control group										
*t*	—	1.91	—	2.37	4.64	4.22	4.10	3.55	4.81	6.09
*p*	ns	0.032	ns	0.012	<.001	<.001	<.001	<.001	<.001	<.001
RTLE group										
*t*	—	2.11	—	—	2.09	—	—	—	3.22	1.95
*p*	ns	.029	ns	ns	.030	ns	ns	ns	.004	.038
LTLE group										
*t*	—	—	—	—	—	—	—	—	—	—
*p*	ns	ns	ns	ns	ns	ns	ns	ns	ns	ns

Note

Results are thresholded at *p* < .05, uncorrected.

Abbreviations: AMY, amygdala; aSTS, anterior superior temporal sulcus; FG, fusiform gyrus; IFG, inferior frontal gyrus; ns, not significant; pSTS, posterior superior temporal sulcus,.

**FIGURE 3:**
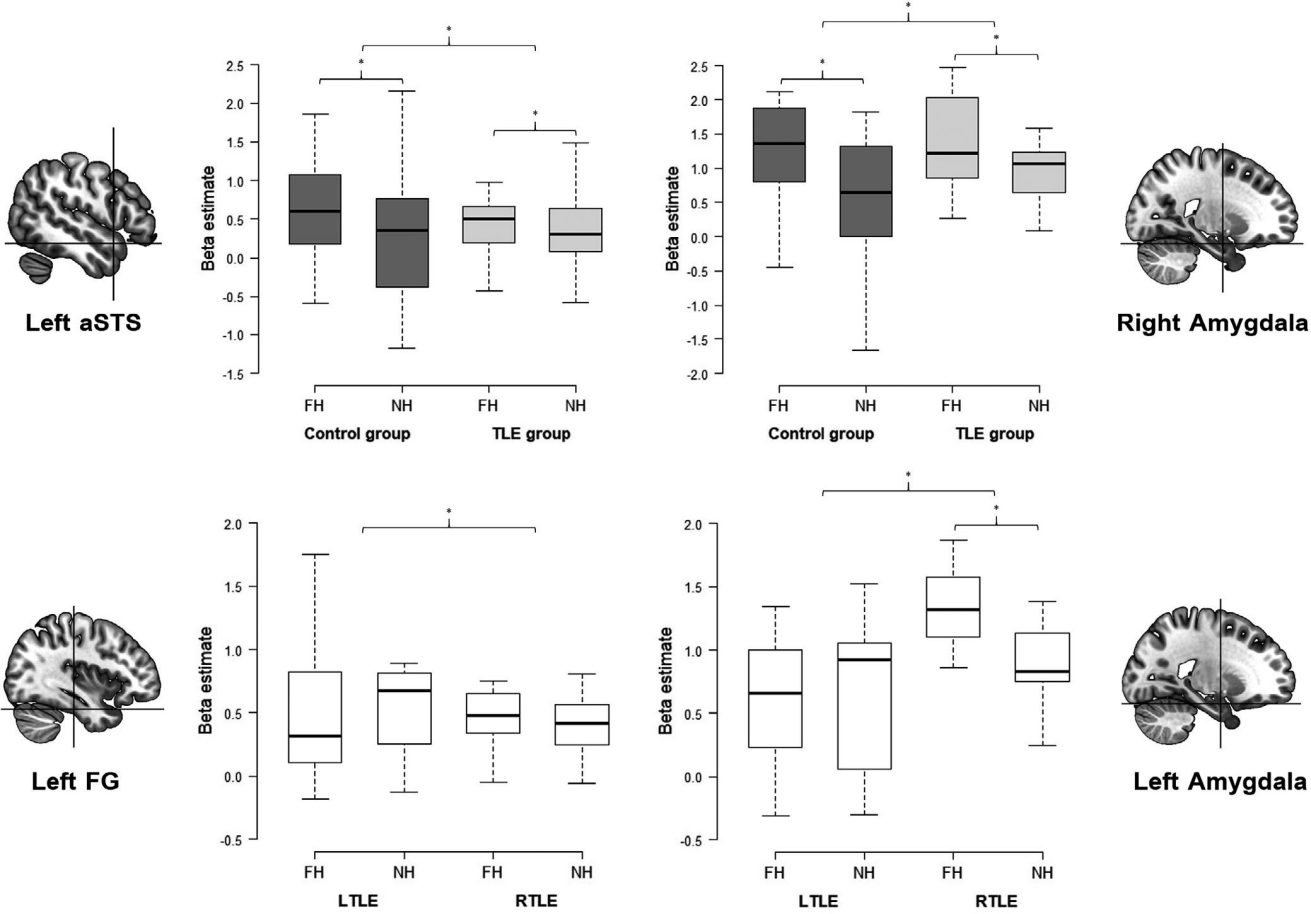
Box plots showing the distribution of beta estimates in response to fearful and neutral human expressions per group and different a priori defined regions of interest. Significant group differences between the control group and the TLE group are displayed in the top panels, whereas significant group differences between people with right TLE and left TLE are displayed in the bottom panels. Whiskers indicate the 25th and the 75th percentile. *p < .05, uncorrected. aSTS, anterior superior temporal sulcus; FG, fusiform gyrus; FH, fearful human expression; LTLE, left temporal lobe epilepsy; NH, neutral human expression; RTLE, right temporal lobe epilepsy; TLE, temporal lobe epilepsy

To test the hypothesis of lower activity in the extended face perception network (i.e., in the a priori defined ROIs) in people with TLE, we compared the response difference between fearful and neutral human expressions in the control group to that in the TLE group. We observed a larger response difference in the right amygdala (*t* = 2.09, *p* =* *.002) and the left aSTS (*t* = 1.71, *p* =* *.048) in controls compared to people with TLE (see Figure 3 for distribution of beta weights per condition and group). For the inverse contrast comparing the response difference in the TLE group to that in the control group, no significant difference between groups was apparent (all *p* >* *.05).

We next compared people with right TLE to those with left TLE. For the right TLE group compared to the left TLE group, we found a larger response difference between fearful and neutral human expressions in the left amygdala (*t* = 1.94, *p* =* *.039) and the left FG (*t* = 1.82, *p* =* *.048; see Figure 3 for distribution of beta weights per condition and group). No difference was found between the two TLE groups, when we compared the activity in the left TLE group in response to fearful and neutral human expressions relative to the right TLE group (all *p* >* *.05).

Regarding analyses with avatar faces, we also compared the response difference between fearful and neutral avatar expressions in the control group to that in the TLE group. No significant response difference was found between the control group and the TLE group in any of the ROIs when comparing fearful and neutral avatar expressions (all *p* >* *.05). Similarly, no response difference was found between the two TLE groups when comparing fearful and neutral avatar expressions (all *p* >* *.05).

### 3.4 Do avatar facial expressions evoke different BOLD responses in the extended face perception network than human facial expressions?

**FIGURE 4:**
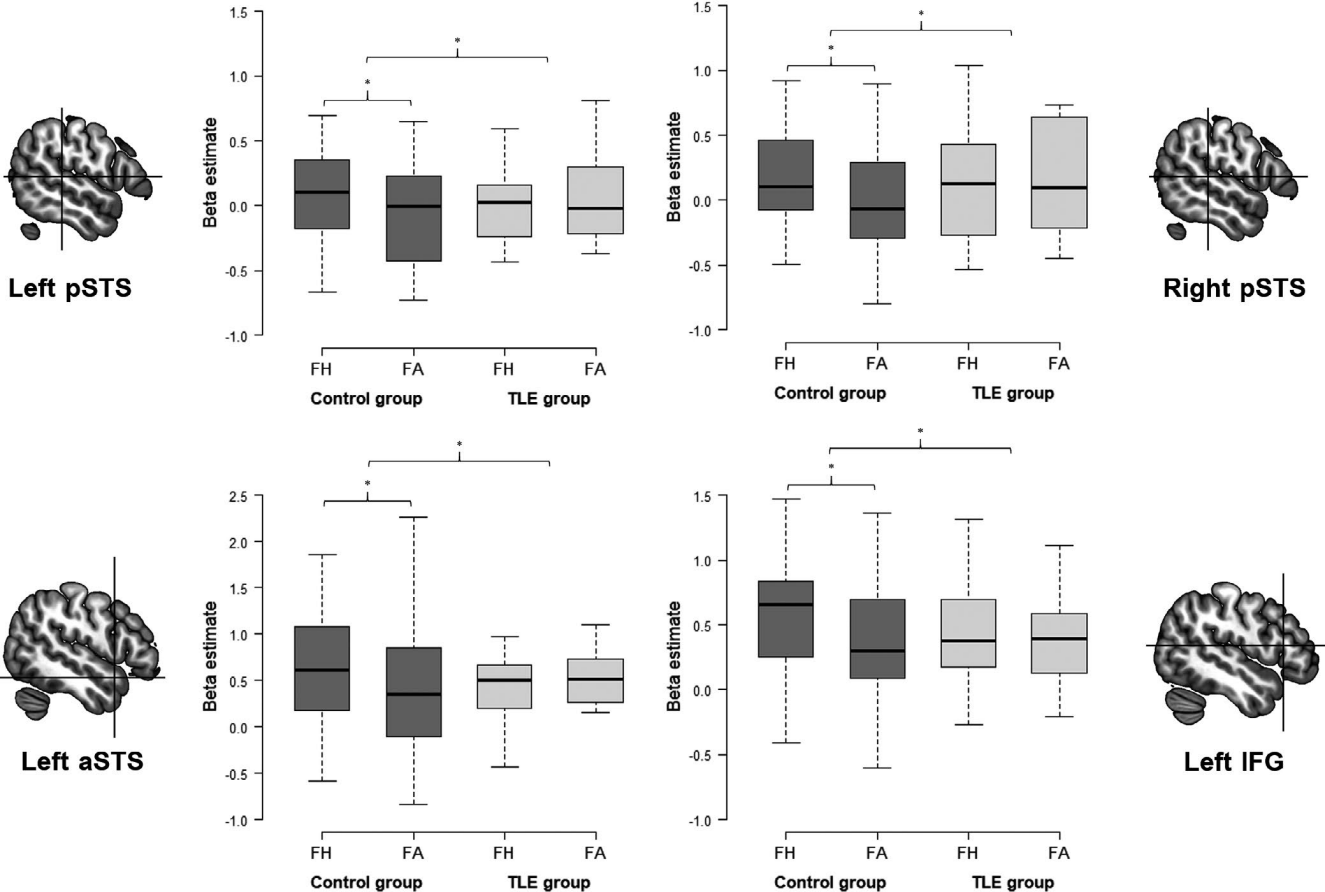
Box plots showing the distribution of beta estimates in response to fearful human and avatar expressions per group and different a priori defined regions of interest. Whiskers indicate the 25th and the 75th percentile. *p < .05, uncorrected. aSTS, anterior superior temporal sulcus; FA, fearful avatar expression; FH, fearful human expression; IFG, inferior frontal gyrus; pSTS, posterior superior temporal sulcus; TLE, temporal lobe epilepsy

When contrasting fearful human versus fearful avatar expressions between groups, we observed a larger response difference for controls in the right and left pSTS, the left aSTS, and the left IFG compared to people with TLE (see Table 3 for between‐group statistics regarding a priori defined ROIs). This indicates that in controls the difference in BOLD response between fearful human and avatar expressions was larger than in people with TLE in almost all the ROIs. This difference between groups was due to comparable responses (i.e., not significantly different) to fearful human and avatar expressions in people with TLE (see Figure 4 for distribution of beta weights per condition and group). The inverted contrast testing for larger response differences in the TLE group compared to the control group was not significant in any of the a priori defined ROIs.

**TABLE 3:** Between‐group comparisons for the contrast human fearful expressions > avatar fearful expressions for each a priori defined region of interest in the extended face perception network

	FG		pSTS		aSTS		IFG		AMY	
	Left	Right	Left	Right	Left	Right	Left	Right	Left	Right
Control > TLE										
*t*	—	—	1.88	2.07	2.53	—	1.85	—	—	—
*p*	ns	ns	.034	.022	.008	ns	.036	ns	ns	ns
RTLE > LTLE										
*t*	—	—	2.26	—	3.56	—	—	—	2.51	—
*p*	ns	ns	.023	ns	.002	ns	ns	ns	.014	ns
LTLE > RLTE										
*t*	—	—	—	—	—	—	—	—	—	—
*p*	ns	ns	ns	ns	ns	ns	ns	ns	ns	ns

Note

Results are thresholded at *p* < .05, uncorrected.

Abbreviations: AMY, amygdala; aSTS, anterior superior temporal sulcus; FG, fusiform gyrus; IFG, inferior frontal gyrus; ns, not significant.; pSTS, posterior superior temporal sulcus.

When comparing people with right TLE and left TLE, the right TLE group showed a larger response difference between fearful human and avatar expressions in the left amygdala, left pSTS, and left aSTS compared to the left TLE group. The left TLE group did not show a larger response difference in comparison to the right TLE group in any of the ROIs.

No significant response difference between any group was found when comparing neutral human and avatar expressions one with each other.

### 3.5 Whole‐brain group comparisons

**FIGURE 5:**
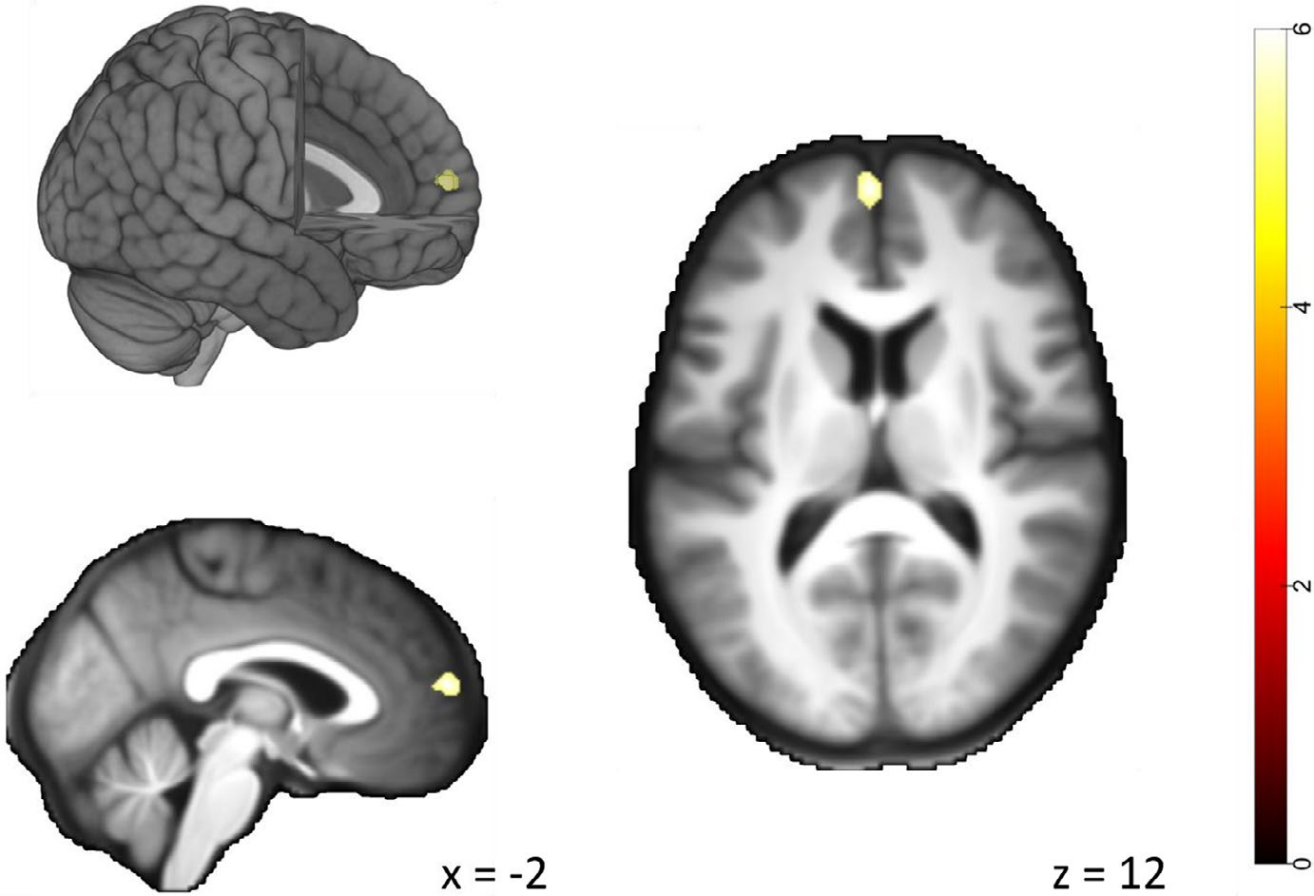
Group‐level statistical parametric map showing the larger response difference between the control group and the TLE group in response to fearful human compared to fearful avatar expressions (voxel‐wise *p*‐FWE < .05). The cluster in the medial prefrontal cortex is shown on the mean anatomical template of the study population (bottom and right image) and on the ‘mni152_2009bet’ template from MRIcroGL (top image)

To determine possible activation differences between people with TLE and controls that arise beyond the extended face perception network, group comparisons were analyzed across the whole brain. This analysis revealed one significant cluster: When comparing fearful human and avatar expressions between the control group and the TLE group, the control group showed a stronger response difference in the medial segment of the left prefrontal cortex (mPFC; MNI *x*, *y*, *z* =* *−2, 60, 12; *t* = 5.98; *k* = 32; *p*‐FWE =* *.008; see Figure 5). This group difference emerged because the activation cluster in the mPFC only occurred in the control group and was absent in the TLE group. Other group comparisons did not reach significance after correction for multiple comparisons (*p*‐FWE >* *.05).

## 4 DISCUSSION

### 4.1 Summary

We investigated whether brain responses to dynamic expressions displayed by human and avatar faces differ between people with TLE and controls. In line with previous research, we were able to demonstrate altered BOLD responses to dynamic human expressions within the face perception network in people with TLE relative to controls. More precisely, people with TLE showed a smaller activation difference between fearful and neutral human expressions in the right amygdala and the left aSTS than controls. When comparing the response difference between fearful and neutral human expressions among people with TLE, we found that the left amygdala and the left FG showed a stronger response difference in people with right TLE compared to those with left TLE. Remarkably, when we compared activity for fearful human and avatar expressions, we found a higher number of significantly different response clusters between groups. Controls showed stronger response differences in the right and left pSTS, the left aSTS, the left IFG, and the left mPFC compared to people with TLE. When investigating response differences between people with right TLE compared to those with left TLE, we observed that the right TLE group showed a stronger response difference between fearful human and avatar expressions contralaterally in the left amygdala, the left pSTS, and the left aSTS.

### 4.2 Altered responses to human facial expressions in temporal lobe epilepsy

In line with our first hypothesis, people with TLE showed an attenuated response difference in the right amygdala between fearful and neutral expressions portrayed by humans compared to controls. Thus, activity in the right amygdala was less increased in people with TLE when observing an emotional expression in another human. This finding is compatible with previous studies that investigated the processing of dynamic human expressions in TLE. This research consistently showed reduced amygdala activity in people with TLE compared to controls (Ives‐Deliperi et al., 2017; Ives‐Deliperi & Jokeit, 2019; Labudda et al., 2014; Schacher, Haemmerle, et al., 2006; Toller et al., 2015). Thus, our results extend earlier evidence to newly developed stimulus material and underline the importance of the right amygdala for the processing of emotional facial expressions.

We also expected people with TLE to show attenuated responses to expressions portrayed by humans in dorsal and ventral areas of the face perception network. However, this hypothesis was statistically confirmed only for the left aSTS showing a smaller response difference between fearful and neutral human expressions in people with TLE compared to controls. The aSTS, as part of the dorsal pathway in the face perception network, is sensitive to dynamic features of faces such as wrinkles and facial motion. This region thus plays a key role in decoding dynamic facial expressions (Duchaine & Yovel, 2015; Haxby et al., 2000; Pitcher et al., 2011). Summarized, this result supports previous studies showing that the epileptogenic network may interfere with (emotional) face processing in people with TLE (Åhs et al., 2014; Riley et al., 2015; Steiger et al., 2017).

Further, people with right TLE showed contralaterally larger response differences between human fearful and neutral expressions in the left amygdala and the left FG relative to people with left TLE. In contrast, there was no observation of larger response differences in left TLE compared to right TLE. The interpretation of this result must be addressed cautiously as previous findings are mixed. Whereas one study also reported stronger amygdala responses in people with right TLE than left TLE (Bonelli et al., 2009), several other studies reported less activity of the amygdala and face‐sensitive areas ipsilateral to seizure onset for right and left TLE (Ives‐Deliperi et al., 2017; Labudda et al., 2014; Schacher, Haemmerle, et al., 2006; Toller et al., 2015). Note, however, that the mentioned studies used different fMRI paradigms either comparing static fearful and neutral expressions (Bonelli et al., 2009) or comparing dynamic fearful expressions to complex landscape scenes (Ives‐Deliperi et al., 2017; Labudda et al., 2014; Schacher, Haemmerle, et al., 2006; Toller et al., 2015).

Compensatory brain activity in people with TLE in the nonaffected, contralateral hemisphere may be associated with larger response differences in right TLE than left TLE (Bettus et al., 2009; Doucet et al., 2013). Due to the preferential role of the right temporal lobe in emotion processing (De Winter et al., 2015; Gainotti, 1972), brain responses during facial emotion processing have been shown to be more affected in people with right TLE than in those with left TLE (Labudda et al., 2014; Steiger et al., 2017). Accordingly, people with right TLE may present stronger compensatory activity in the contralateral hemisphere than those with left TLE, which may account for the group differences found. The explanation would also be consistent with the larger activation difference in people with right TLE in the left amygdala, left pSTS, and left aSTS in response to fearful human and avatar expressions compared to people with left TLE.

### 4.3 Altered responses to avatar facial expressions in temporal lobe epilepsy

Corresponding to our second hypothesis, we found larger response differences for controls between fearful human and avatar expressions in the pSTS and aSTS compared to people with TLE. More precisely, no significantly different responses were found in people with TLE between fearful human and avatar expressions in dorsal temporal cortex. This result is comparable to a previous study examining brain responses in people with TLE after resection of the anterior temporal lobe (Åhs et al., 2014). In this study, individuals who underwent resection showed reduced responses in the pSTS to fearful human expressions compared to controls. Similar reduced responses of the pSTS to fearful human expressions were observed in people with TLE before resection (albeit not statistically significant; Riley et al., 2015). These results support the notion that structural and functional changes in the (mesial) temporal lobe affect brain functions in structurally intact face processing areas (Vuilleumier & Pourtois, 2007; Vuilleumier et al., 2004). Additionally, it gives support to the modulatory effect of mesial temporal areas, particularly the amygdala, on dorsal temporal cortex during (emotional) face processing (Furl et al., 2013). This highlights the influence of emotion on perceptual, cognitive, and motor responses to dynamic facial expressions (Sato et al., 2017; Vuilleumier & Pourtois, 2007).

The striking finding of our study was larger response differences in controls between fearful human and avatar expressions in frontal areas such as the IFG and the mPFC relative to people with TLE. We are the first to report activation differences in frontal areas during facial emotion processing between people with TLE and controls. This coincides with findings that altered functions in the mesial temporal lobe affect activity and connectivity throughout the entire brain (Ives‐Deliperi & Jokeit, 2019; Jokeit et al., 1997; Riley et al., 2015; Steiger et al., 2017). Moreover, this altered brain activity may not only be related to facial emotion processing, but to other socio‐cognitive processes such as self‐other distinction associated with the IFG (Sinigaglia & Rizzolatti, 2011), as well as mentalizing, perspective taking, or self‐referential processing related to the mPFC (Lieberman et al., 2019; Van Overwalle, 2009). Support for this hypothesis comes from a study investigating BOLD responses in people with TLE during processes related to the attribution of mental states. This study showed that people with TLE exhibited limited neural responses compared to controls when they observed animations of interactions that involved the attribution of mental states (Hennion et al., 2016).

### 4.4 Limitations and future directions

Our study is the first to apply dynamic avatar stimuli in the research on facial emotion processing in epilepsy. Understandably, we want to discuss certain limitations. First, the low sample size in the right and left TLE groups (*n* = 7 each) may have limited the statistical power to detect small differences between the two groups. Second, we report ROI results that are not corrected for multiple comparisons (i.e., not corrected for the total number of regions in the ROI analysis). As Bonferroni's adjustment for multiple comparisons is often too conservative (Field, 2009), we decided to report this exploratory, but initial evidence concerning the processing of human and avatar expressions in individuals with and without TLE.

Being the first study to apply dynamic avatar stimuli, we focused on fearful expressions given their evolutionary importance and their frequent use in research in TLE (Adolphs, 2008; Ives‐Deliperi & Jokeit, 2019). Moving on from this, future studies could incorporate expressions of additional emotions. Notably, this requires software solutions that enable us to render realistic emotional expressions with even subtle differences such as expressions of fear and surprise. Additionally, future studies may investigate whether processing differences between individuals with and without TLE also translate onto behavior toward avatars. This is highlighted by the fact that behavioral impairments in human emotion recognition in people with TLE are often subtle despite extensive structural and functional changes on a neural level (Monti & Meletti, 2015). Considering this, future studies may clarify whether tasks with avatars may be implemented for the clinical assessment of emotion perception in individuals with TLE.

## 5 CONCLUSIONS

Our results show that the neural processing of human and avatar facial expressions differs between individuals with and without TLE in (a) dorsal temporal and inferior frontal cortex sensitive to dynamic facial information and (b) medial prefrontal cortex associated with processes related to the self and others such as mentalizing, perspective taking, or self‐referential processing. Further, our findings support previous studies showing that BOLD activity in the amygdala and the face perception network is altered in individuals with TLE—in response to human as well as to avatar faces. Thus, in individuals with TLE, the influence of altered BOLD activity in the temporal lobe should also be extended to artificial facial expressions. Is this altered BOLD activity an expression of the underlying pathology or a response of a network that can overcome impairment due to temporal brain lesions? Since previous studies have shown that comparable changes in BOLD activity, including connectivity, are associated with impairments in human emotion recognition in people with TLE, but not necessarily with other forms of epilepsy, we can now convincingly argue that it is necessary to study the social domains of patients' behavior when using avatars (Broicher et al., 2012; Ives‐Deliperi & Jokeit, 2019; Labudda et al., 2014; Steiger et al., 2017; Toller et al., 2015). Considering the increased use of avatars in digital applications and remote communication technologies, this study highlights the importance of investigating neural and behavioral responses to computer‐generated characters in samples with neurological conditions as they may respond differentially to our new socio‐digital environment.

## CONFLICT OF INTEREST

The authors declare no conflict of interest.

### PEER REVIEW

The peer review history for this article is available at https://publons.com/publon/10.1002/brb3.2140.

## Supporting information

Video S1

Video S2

Video S1 and S2

## Data Availability

The data that support the findings of this study are available on request from the corresponding author. The data are not publicly available due to privacy or ethical restrictions.
