# Supplementary material for: Temporal lobe epilepsy alters neural responses to human and avatar facial expressions in the face perception network
Source: Brain Behav. 2021 May 5;11(6):e02140. doi: 10.1002/brb3.2140 (PMC8213650; doi:10.1002/brb3.2140)
Supplement: Supplementary file 3 — Video S1 and S2 [file BRB3-11-e02140-s002.docx]

*Video S1 and S2.* Example videos showing fearful expressions displayed by a human and an avatar face.
